# Prostaglandin F2-alpha receptor (FPr) expression on porcine corpus luteum microvascular endothelial cells (pCL-MVECs)

**DOI:** 10.1186/1477-7827-5-31

**Published:** 2007-07-20

**Authors:** Augusta Zannoni, Chiara Bernardini, Tommaso Rada, Luciana A Ribeiro, Monica Forni, Maria L Bacci

**Affiliations:** 1 Department of Veterinary Morphophysiology and Animal Production, DIMORFIPA, Ozzano Emilia 40064, University of Bologna, Italy; 2 Department of Polymer Engineering, University of Minho 4710-057 Braga, Portugal

## Abstract

**Background:**

The corpus luteum (CL) is a transient endocrine gland and prostaglandin F2-alpha is considered to be the principal luteolysin in pigs. In this species, the in vivo administration of prostaglandin F2-alpha induces apoptosis in large vessels as early as 6 hours after administration. The presence of the prostaglandin F2-alpha receptor (FPr) on the microvascular endothelial cells (pCL-MVECs) of the porcine corpus luteum has not yet been defined. The aim of the study was to assess FPr expression in pCL-MVECs in the early and mid-luteal phases (EL-p, ML-p), and during pregnancy (P-p). Moreover, the effectiveness of prostaglandin F2-alpha treatment in inducing pCL-MVEC apoptosis was tested.

**Methods:**

Porcine CLs were collected in the EL and ML phases and during P-p. All CLs from each animal were minced together and the homogenates underwent enzymatic digestion. The pCL-MVECs were then positively selected by an immunomagnetic separation protocol using Dynabeads coated with anti-CD31 monoclonal antibody and seeded in flasks in the presence of EGM 2-MV (Microvascular Endothelial Cell Medium-2). After 4 days of culture, the cells underwent additional immunomagnetic selection and were seeded in flasks until the confluent stage.

PCR Real time, western blot and immunodetection assays were utilized to assess the presence of FPr on pCL-MVEC primary cultures. Furthermore, the influence of culture time (freshly isolated, cultured overnight and at confluence) and hormonal treatment (P4 and E2) on FPr expression in pCL-MVECs was also investigated. Apoptosis was detected by TUNEL assay of pCL-MVECs exposed to prostaglandin F2-alpha.

**Results:**

We obtained primary cultures of pCL-MVECs from all animals. FPr mRNA and protein levels showed the highest value (ANOVA) in CL-MVECs derived from the early-luteal phase. Moreover, freshly isolated MVECs showed a higher FPr mRNA value than those cultured overnight and confluent cells (ANOVA). prostaglandin F2-alpha treatment failed to induce an apoptotic response in all the pCL-MVEC cultures.

**Conclusion:**

Our data showing the presence of FPr on MVECs and the inability of prostaglandin F2-alpha to evoke an in vitro apoptotic response suggest that other molecules or mechanisms must be considered in order to explain the in vivo direct pro-apoptotic effect of prostaglandin F2-alpha at the endothelial level.

## Background

The corpus luteum (CL) is a transient endocrine gland essential for the regulation of ovarian cycles as well as for establishing and maintaining pregnancy. Prostaglandin F_2α _(PGF_2α_) is considered the principal luteolysin in domestic animals and acts on target cells [1] by means of a specific plasma membrane-associate receptor, prostaglandin F2-alpha receptor (FPr). The FPr was detected on steroidogenic luteal cells [2-5] but its presence on other target cells could not be excluded.

Our previous data [6] showed that, in a porcine CL regression model, cloprostenol (a PGF_2α _synthetic analogue) first induces apoptosis of luteal endothelial cells then apoptosis of steroidogenic cells and suggest that the structural regression of the CL may begin with an early response of the endothelial compartment to PGF_2α_. This hypothesis relies on the assumption that FPrs are present on endothelial cells (ECs). The expression of the FPr was described by RT-PCR in bovine ECs derived from CLs [7]; on the contrary, FPr mRNA has been identified only on swine steroidogenic cells by an in situ study [8]. Therefore, to date, the presence of the FPr on luteal ECs is not fully clarified, perhaps due to the different techniques utilized.

The porcine CL is not sensitive to the luteolytic stimulus until day 12 of the cycle [9-11]. Luteal PGF_2α _binding sites increase during CL formation reaching a maximum on day 13 of the oestrous cycle, concurrently with the onset of luteal sensitivity to PGF_2α _[3,12]. Limited data exist confirming that an FPr increase is responsible for the acquisition of luteolytic sensitivity.

The importance of the interaction between luteal cells and ECs was demonstrated both in *in vivo *and *in vitro *experiments in bovine and swine species [13-17].

The interest in EC biology increased in parallel with the optimization of successful protocols for their isolation and culture, allowing the study of EC involvement in many different physiological or pathological processes. EC isolated from large vessels or from tissue microvasculature show a morphological heterogeneity [18,19] and significant functional differences [20]. Additional differences have been observed among EC isolated from microvessels of different organs [21,22] as well as from different regions of the same organ [23-25]. These differences are mainly due to the local micro-environment (e.g., the presence of matrix proteins, soluble cytokines, growth factors and paracrine signals) and to cell-to-cell communication.

In this context, the isolation and characterization of pCL-MVECs may lead to a better insight into endocrine gland physiology and contribute to explaining some physiological differences during the CL lifespan.

Many studies have been focused on the CL-MVEC isolation in different species [23,26-31] except swine where MVEC isolation has been described only in the fetal organs [32] and the heart [33].

In order to obtain pure MVEC cultures from CLs at different stages (early and mid-luteal phases and during pregnancy), the initial aim of the present study was to define a reliable isolation method. Thus, our subsequent aims were to investigate the presence of the FPr, both at the mRNA and the protein level, on pCL-MVEC and to verify if PGF_2α _treatment is effective in inducing pCL-MVEC apoptosis.

## Materials and methods

### pCL-MVEC culture set-up

#### Animals and corpora lutea collection

Twelve prepubertal Large White gilts (94 ± 1.47 Kg body weight) were injected i.m. with 1250 iu (international units) of equine chorionic gonadotropin (eCG; Folligon-Intervet, Holland) and, 60 h later, with 750 iu of human chorionic gonadotropin (hCG; Corulon-Intervet) to synchronize ovulation. The animals were randomly assigned to three groups (n. = 4). Four animals were artificially inseminated at 40/44 h post-hCG treatment and pregnancy was determined after 30 days by ultrasonography. The ovaries were surgically removed from all animals: 4 at the early luteal phase (EL-p: 6 days after ovulation), 4 at the mid-luteal phase (ML-p: 12 days after ovulation) and 4 during the pregnancy phase (P-p: 60 days of pregnancy).

All animals were housed and used according to EEC animal care guidelines. The experimental procedures had previously been submitted to and approved by the Ethical Committee of Bologna University.

#### Isolation and culture conditions of Microvascular Endothelial Cells (MVECs)

The ovaries were immediately taken to the laboratory in ice-cold DPBS (Dulbecco Phosphate Buffer Saline; Cambrex Bio Sciences, Verviers, Belgium) and washed several times in sterile DPBS; the corpora lutea (CLs) were then isolated under sterile conditions.

All CLs from each animal (14.1 ± 5.9) were cut into small fragments (~1 mm^3^) with a razor blade and minced together with a pestle and mortar. Small aliquots (100 μg) of homogenate were immediately frozen in liquid nitrogen for RNA extraction, and protein and progesterone (P_4_) analyses. The remaining homogenate was washed (3 times) by adding sterile DPBS (1:3, w:v) and centrifuged (500 × g, 5 min). The final pellet was resuspended in sterile DPBS containing collagenase (2 mg/ml; type IA [420 U/mg]; Sigma Chemical Company, St. Louis, MO, USA), BSA (4 mg/ml; Fraction V, Sigma) and DNase I (0.02 mg/ml; [1.87 U/mg]; Sigma). After 20 min on a rocking platform (200 rpm) at 37°C, enzymatic digestion was stopped by adding FBS (Foetal Bovine Serum, Gibco-Invitrogen, Paisley, UK) (1:1, v/v); the suspension was filtered (pore sizes: 280 μm; Sigma) and placed on ice, then centrifuged (800 × g, 20 min) and resuspended in DPBS-BSA (0.1%; w/v) at a final concentration of 5 × 10^5 ^cells/ml (Thoma chamber counting). The pCL-MVECs were then positively selected by an immunomagnetic separation protocol using Dynabeads^® ^coated with anti-CD31 monoclonal antibody (Invitrogen), according to the manufacturer's instructions. Briefly, the cells were mixed with Dynabeads^® ^to give a ratio of 5 × 10^5 ^cells/4 × 10^8 ^beads/ml and were then incubated for 20 min at 4°C on a rocking platform.

After incubation, the suspension was diluted (1:3, v/v) with DPBS-BSA (0.1%), endothelial cells were then exposed to Magnetic Particle Concentrators (DYNAL MPC^®^, Invitrogen) for 2 min and washed five times in DPBS-BSA.

The last resuspension was carried out in EGM™2-MV medium (Microvascular Endothelial Cell Medium-2, Cambrex); cell viability was determined using the Trypan Blue exclusion test and viable cell density was calculated using a Thoma chamber.

The pCL-MVECs (3 × 10^5 ^viable cells/flask) were then placed in a T-25 tissue culture flask (Falcon, Beckton-Dickinson, Franklin Lakes NJ, USA) in EGM™2-MV supplemented with BulletKit^® ^(Cambrex) in an humidified atmosphere at 5% CO_2 _(38.5°C). After 4 days of culture, the cells were trypsinized (Trypsin-EDTA solution, Sigma) for 6 min at 37°C, further selected with Dynabeads^® ^and seeded again in EGM™2-MV supplemented with BulletKit^®^. At the 5^th ^day of culture, the medium was substituted by Human Endothelial-SFM (HE-SFM; Gibco-Invitrogen) supplemented with 15% FBS (Gibco-Invitrogen) and 1% antibiotic/antimicotic solution (Gibco-Invitrogen); the medium was changed every 24 h until confluence. The cultures were observed daily using phase-contrast inverted microscopy (Nikon Eclipse TS100).

In addition, freshly isolated (dispersed) and overnight (ON) cultured of ML-p pCL-MVECs were recovered; in order to test the microenvironmental effects of steroid hormones, the ML-p pCL-MVECs were cultured in complete HE-SFM medium with and without hormones (P_4_, 10^-6 ^M; Sigma Cod. P0130; 17_β_-estradiol [E_2_], 10^-8 ^M; Sigma Cod. E8875) until confluence.

Confluent cultures of porcine Aorthic Endothelial Cells (pAEC) (8^th ^passage) were assessed [34] at the same time.

#### Progesterone assay

Tissue P_4 _levels were determined as previously described by Ribeiro *et al. *[35]. A small amount of frozen CL homogenate (for each animal) was further homogenised by Ultra Turrax in PBS (0.1 g/ml) in an ice bath. An aliquot (20 μl) was extracted with petroleum ether (1 h at RT on a rotary shaker) and centrifuged. The ether was then collected and dried under a N_2 _stream. The dried ether extract was resuspended in PBS (1 ml), diluted 1:50 and an aliquot of 50 μl was then assayed using a validated Radio Immuno Assay (RIA) [36]. The P_4 _results are expressed as ng/mg tissue. The intra- and inter-assay coefficients of variation were 5.2 % and 9 %, respectively.

#### Characterization of pCL-MVEC cultures

Cells were grown in slide chambers (8 well Culture Slide, Falcon™, Becton-Dickinson) for 24 h and immunostained for endothelial cell markers (factor VIII, CD31). The slides were fixed with ethanol/acetic acid (2:1), washed with PBS three times and blocked with 10% (v/v) FBS in PBS for 1 h at RT. Primary antibodies, 1:200 rabbit anti-human Factor VIII (A0082, Dako A/S, Glostrup, Denmark) or 1:100 mouse anti-porcine CD31 (MCA1746, Serotec LTD, Oxford, UK), were added for 1 h at RT; then, after several washings with PBS, secondary fluorescein isothiocyanate (FITC)-conjugated antibodies were added (1:800 in DPBS). Negative controls without primary antibodies were performed.

The cells were counterstained with propidium iodide (PI) and examined under an epifluorescence microscope (Eclipse E600, Nikon, Japan) with fluorescein (FITC) and tetramethylrhodamine (TRITC) filters using a Nikon digital camera. A minimum of 100 cells was evaluated in each experimental sample.

To evaluate the presence of contaminant steroidogenic luteal cells, a PCR Real time amplification for Steroidogenic acute regulatory protein (StAR) [37] was conducted on pCL-MVEC cultures, as described below.

### Identification of FPr

#### RNA extraction and Real time PCR

Total RNA was extracted from endothelial cells (pCL-MVECs and pAECs) using RNeasy Mini Kits 50 (Qiagen Sciences Inc, MD, USA) and was treated with an RNase-free DNase set (Qiagen) according to the manufacturer's instruction. Total RNA was extracted from tissue CL homogenates using a TRIZOL isolation reagent (Invitrogen) according to the manufacturer's instructions.

Purified RNAs were resuspended in 25 μl of RNAse-free water and spectrophotometrically quantified (A260 nm); their quality was checked by gel electrophoresis on 2% agarose gel. One microgram of total RNA was reverse-transcribed to cDNA using an iScript cDNA Synthesis Kit (Bio-RAD Laboratories Inc., California, USA) for a final volume of 20 μl. Transcription reactions without reverse transcriptase were performed in order to check for possible DNA contamination.

Swine primers (FPr, StAR, HPRT [Hypoxanthine phosphoribosyltransferase]) were designed to span one intron, to avoid genomic DNA amplification, using Beacon Designer 2.07 Software (Premier Biosoft International, Palo Alto, CA, USA). Primer sequences, expected PCR product lengths and sequence accession numbers are shown in Table 1.

**Table 1:** Forward and reverse primer sequences, RT-PCR product length and accession number (Acc.No.) in the EMBL database.

Primer	Sequence (5'-3')	Product Length (bp)	Acc. No.
FPr	For.: TCAGCAGCACAGACAAGG Rev.: TTCACAGGCATCCAGATAATC	151	AY043485
StAR	For.: GGACATCCTCAGCAACCAG Rev.: GTCCACCACCACCTCCAG	121	U53020
HPRT	For.: GGACAGGACTGAACGGCTTG Rev.: GTAATCCAGCAGGTCAGCAAAG	115	AF143818

Real-time quantitative PCRs were performed in the iCycler Thermal Cycler (Bio-RAD) using SYBR green I detection. A master-mix of the following reaction components was prepared to indicate end-concentrations: 0.5 μl forward primer (0.2 μM), 0.5 μl reverse primer (0.2 μM), 9 μl water and 12.5 μl IQ SYBR Green BioRad Supermix (Bio-RAD Laboratories Inc.). cDNA (2.5 μl) was added to the master mix (22.5 μl). All samples were assayed in duplicate. The real-time PCR protocol employed was the following: initial denaturation for 3 min at 95°C, 40 cycles at 95°C for 15 sec and 60°C for 30 sec, followed by a melting step from 55° to 95°C with a rate of 0.5°C/s. The housekeeping gene HPRT data were used to normalize the RNA amount in all samples.

Real-time efficiency for each primer set was acquired by amplification of a standardized cDNA dilution series. The specificity of the amplified PCR products was verified by melting curve analyses and an agarose gel electrophoresis run. The housekeeping gene HPRT was used to normalize the amount of RNA. FPr mRNA expression was calculated as ΔC_T _(HPRT C_T _- FPr C_T_) [38] for all samples.

#### Protein extraction and Western Blot

The protein contents of CL homogenates and endothelial cell lysates (sonication in PBS, 1 × 10^6 ^cells/200 μl) were determined by a protein assay kit (Sigma). Protein aliquots (20 μg) were separated on NuPage 10 % Bis-Tris Gel (Invitrogen) for 50 min at 200 V and were then electrophoretically transferred onto a nitrocellulose membrane. The blots were washed in PBS, and protein transfer was checked by staining the nitrocellulose membranes with 0.2 % (w/v) Ponceau Red and gels with Comassie Blue. Non-specific protein binding on the nitrocellulose membrane was blocked with 5 % (w/v) milk powder in PBS-T20 (phosphate buffer saline – 0.1 % (v/v) Tween-20) for 1 h at RT. The membranes were then incubated with a 1:500 dilution of a rabbit anti-FPr (NLS 1049, Novus Biologicals Inc, Littleton, CO, USA) in TBS-T20 (20 mM Tris-HCl, pH 7.4; 500 mM NaCl; 0.1 % Tween-20) with 0.5 % milk overnight (O/N) at 4°C. After several washings with PBS-T20, the membranes were incubated with the secondary biotin-conjugated antibody (1:80 000) and then with a 1:1000 dilution of an anti-biotin horseradish peroxidase (HRP)-linked antibody. Western blots were developed using a chemiluminescent substrate (Pierce Biotechnology Inc, Rockford, IL, USA) according to the manufacturer's instructions. The membranes were stripped and reprobed by rabbit anti-HPRT (1:250; FL218, Santa Cruz Biotechnology Inc, Santa Cruz CA, USA) in order to normalize the results. The relative protein content was determined by the density of the resultant bands and expressed in arbitrary units (AU) relative to the HPRT content, using Quantity One Software (Bio-Rad).

#### Immunofluorescent staining

The immunolocalization of the FPr in pCL-MVEC and pAEC cultures was performed by indirect immunofluorescence. Cells grown in slide chambers for 24 h were fixed with acetone and methanol (1:1; v/v) for 10 min at -20°C and washed several times with PBS. To better identify the FPr at the plasma membrane level, immunofluorescent staining was also performed without previous sample fixation. Slides were blocked with 10 % FBS in PBS for 1 h at RT and incubated O/N at 4°C with anti-FPr diluted 1:100 in 10 % FBS in PBS or without a primary antibody (negative control). After several washings in PBS, an FITC-conjugated secondary antibody was added (1:800 at RT for 1 h).

The cells were then counterstained with PI and examined under an epifluorescence microscope equipped with appropriate filters. A minimum of 100 cells were evaluated for each experimental sample.

### PGF_2α _treatment of pCL-MVECs

pCL-MVECs were grown to confluence (70–80 %) in 8 well slide chambers in HE-SFM (15 % FBS) and then exposed to 10 μg/ml PGF_2α _(P5069; Sigma) in HE-SFM (1 % FBS) for 6, 12, and 24 hours. pAEC cultures were also treated with PGF_2α _as well as with LPS (10 μg/mL for 24 h; E. coli 055:B5, Sigma) as positive controls. Apoptosis was determined by the ApopTag Fluorescein in situ apoptosis detection Kit (S7110, Intergen Company, 10577 NY, USA), according to the manufacturer's instructions. The cells were fixed in 1% paraformaldehyde in PBS, post-fixed in precooled ethanol:acetic acid (2:1, v:v), then incubated with dTT enzyme for digoxigenin dNTP incorporation (30 min at 37°C) and, after several washings with DPBS, finally incubated with anti-digoxigenin-fluorescein antibody (30 min at RT in the dark). The cells were counterstained with PI and observed under an epifluorescence microscope. A minimum of 200 cells were evaluated for each experimental point.

### Statistical analysis

Data were analyzed by one-way analysis of variance (ANOVA); the significant differences were analyzed by the Duncan's test (SPSS Inc, Chicago, IL, USA). A probability of P < 0.05 was considered significant. Data represent mean ± SD (n = 4).

## Results

### Assessment of pCL-MVEC cultures

The functional stages of isolated corpora lutea were confirmed by tissue P_4 _concentrations (26.78 ± 4.2, 48.62 ± 4.1 and 90.43 ± 1.81 ng/mg at EL-, ML- and P-phases, respectively).

We were able to obtain primary cultures of pCL-MVECs from all animals. The primary cultures reached confluence (about 10^6 ^cells/flask) in 7.6 ± 2.7 days (min 4, max 12); all cultures derived from EL-p CLs reached confluence faster (5 ± 1 days) than ML-p- and P-p-MVECs.

**Figure 1:**
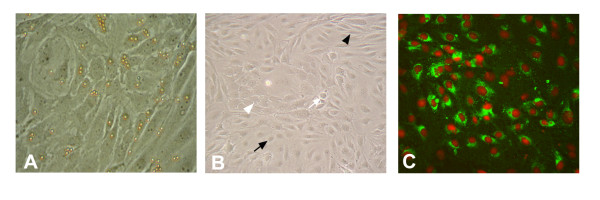
Representative images of mid-uteal-phase porcine corpus luteum-microvascular endothelial cell (ML-p pCL-MVEC) cultures. A) pCL-MVECs after 2 days of culture with beads still attached (phase contrast microscope ×200). B) the pCL-MVEC monolayer at confluence (day 7) (phase contrast microscope ×100). Different endothelial cell types are shown: spindle-shaped cells (*black arrowhead*), cobble-stone cells (*white arrow*), polygonal-opaque cells (*black arrow*), phase-dense cells (*white arrowhead*). C) Immunostaining for FVIII antigen (epifluorescence microscope ×100).

pCL-MVEC cultures showed the typical heterogeneous morphologies already described for bovine CL-MVEC [23,39]. Cobble-stone shaped, polygonal opaque, spindle-shaped and phase-dense MVEC phenotypes were observed (Fig. 1A, B). No evidence of contaminating cells was observed

### Characterization of pCL-MVEC cultures

Identification of the endothelial origin was confirmed by CD31 and factor VIII immunofluorescence. As expected, due to the isolation method used, all cells were positively stained for CD31. The factor VIII distribution varied among MVEC phenotypes, as reported on bovines [30], exhibiting a sometimes perinuclear, diffuse or granular cytoplasmatic pattern while some pCL-MVECs were negative (Fig. 1C). No fluorescence was detected in the absence of a primary antibody.

StAR mRNA was not detectable in any pCL MVECs.

### Real time quantification of the FPr

FPr mRNA expression was calculated as ΔC_T _(HPRT C_T _- FPr C_T_). As the HPRT C_T _were comparable among samples, increased ΔC_T _values represent an increase in FPr mRNA expression.

**Figure 2:**
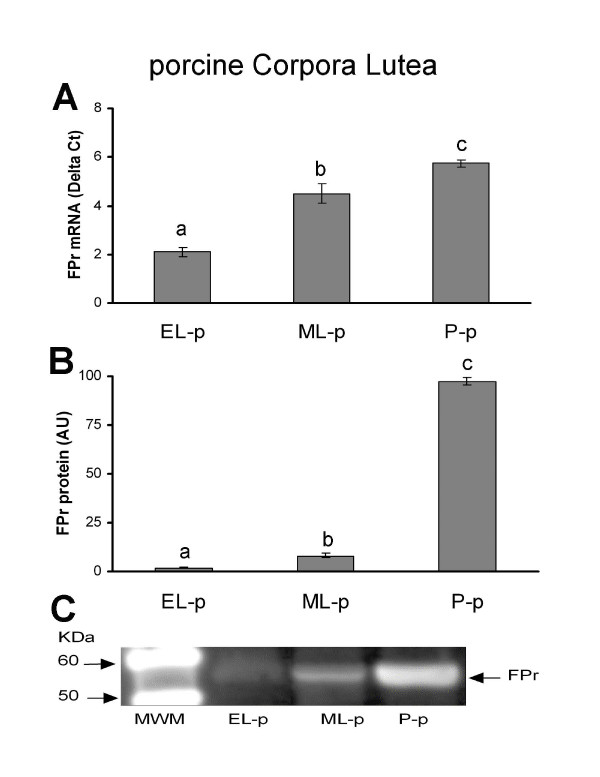
Presence of FPr in porcine corpora lutea (pCLs) at the early-luteal phase (EL-p), the mid-luteal phase (ML-p) and during pregnancy (P-p). A) Relative FPr mRNA expression. The results are presented as Delta Ct (HPRT C_t _- FPr C_t_). B) FPr protein content. The results are presented as AU (arbitrary units). Data represent mean ± SD. Different letters indicate statistically significant differences (P < 0.05). C) Representative Western blotting of FPr.

**Figure 3:**
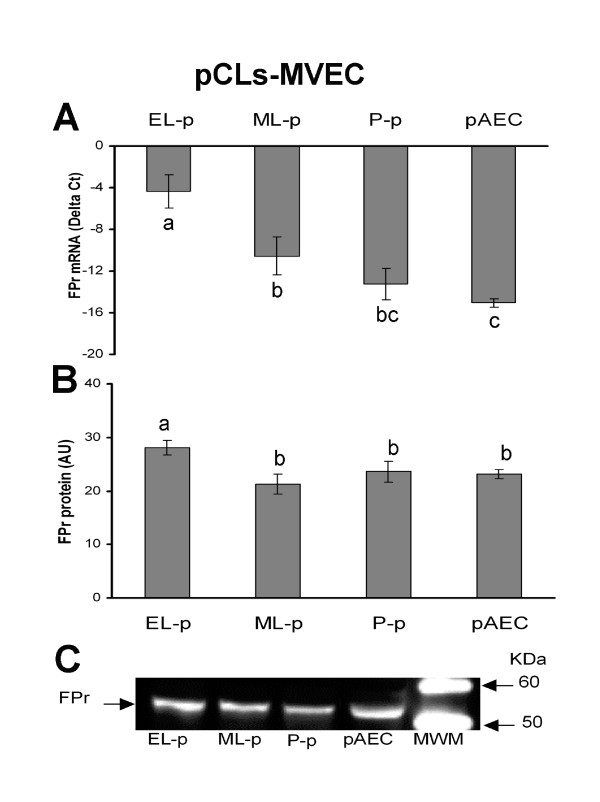
Presence of FPr in porcine corpora lutea-derived microvascular endothelial cells (pCL-MVECs) and porcine Aortic Endothelial Cells (pAECs). A) Relative FPr mRNA expression. The results are presented as Delta Ct (HPRT C_t _- FPr C_t_). B) FPr protein content. The results are presented as AU (arbitrary units). Data represent mean ± SD. Different letters indicate statistically significant differences (P < 0.05). C) Representative Western blotting of FPr.

FPr mRNA expression levels in CL homogenates and in pCL-MVEC confluent cultures are shown in Fig. 2 and Fig. 3, respectively.

The relative amount of FPr mRNA in luteal tissue significantly increased from EL- to ML-, reaching a maximum in the P-phase (Fig. 2A). FPr mRNA was detectable in all pCL-MVEC cultures, showing the highest expression in EL-p derived MVECs; FPr mRNA was also detectable in pAECs (Fig. 3A).

**Figure 4:**
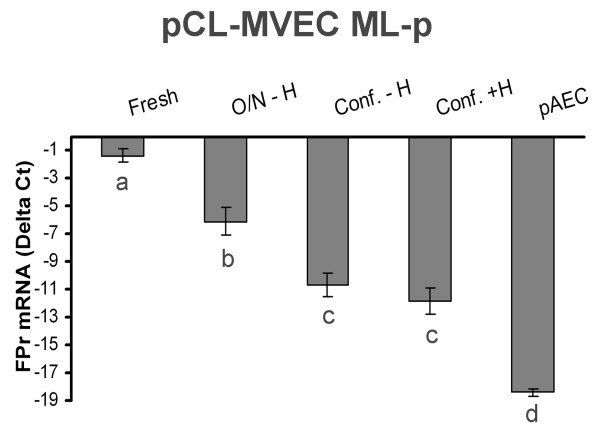
FPr mRNA expression in porcine corpora lutea-microvascular endothelial cells (pCL-MVECs) freshly isolated (Fresh) and cultured for different times (overnight-O/N; confluence-Conf.) and in porcine Aortic Endothelial Cells (pAECs). The confluent stage of the MVECs was reached in the presence (+) or absence (-) of hormonal treatment (H = P_4 _[10^-6^M] + E_2 _[10^-8 ^M]). The results are presented as Delta Ct (HPRT C_t _- FPr C_t_). The data represent mean ± SD. Different letters indicate statistically significant differences (P < 0.05).

ML-p pCL-MVECs analysed at different culture times showed that FPr mRNA was highly expressed in freshly isolated ML-p pCL-MVEC then the expression was significantly reduced after an ON culture and reached the minimum level at confluent stage. The hormonal treatment had no regulative effect on the expression of FPr mRNA expression (Fig 4).

### Immunodetection of FP receptor

Western blot analysis evidenced a band of molecular weight (~55 KDa) in both CLs and endothelial cells (Fig. 2C; Fig. 3C). In luteal tissues, the FPr increased from the EL- to the ML-phase, reaching the highest level during pregnancy (P-p) (Fig. 2B). Similar levels of FPr in ML- and P-phase derived pCL-MVECs and in pAECs were observed; EL-p-derived pCL-MVECs showed a slight but significant increase in the FPr level (Fig. 3B).

**Figure 5:**
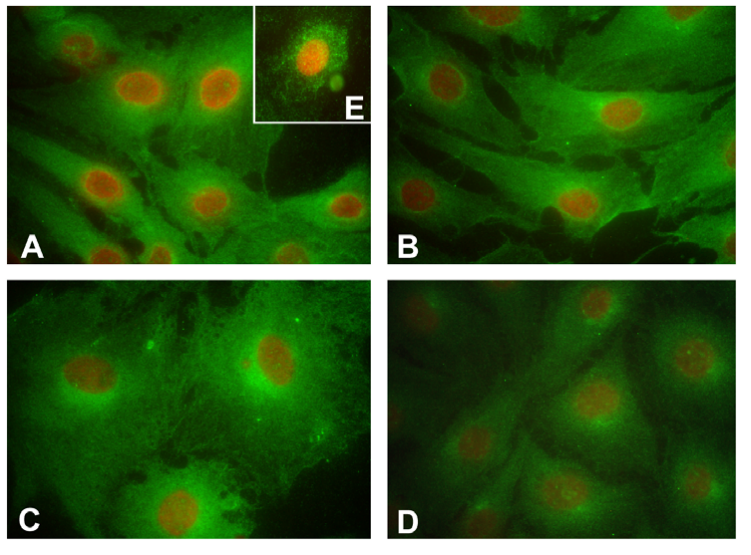
Representative FPr immunofluorescent staining on porcine corpus luteum-microvascular endothelial cells (pCL-MVECs) isolated from the early luteal phase (EL-p) (A), the mid-luteal phase (ML-p) (B) and during pregnancy (P-p) (C) (×400). D) porcine Aortic Endothelial Cell (pAEC) culture (x400). Representative immunostaining of the FPr on non-fixed EL-p pCL-MVECs is shown (E).

The FPr was diffusely distributed in the cytoplasm of all pCL-MVECs and in pAECs (Fig. 5A–D). Moreover, P-p pCL-MVECs showed a clear perinuclear labeling pattern (Fig. 5C). Positive staining was also observed along the plasma membrane and was more evident in non-fixed cells (Fig. 5E). No fluorescence was detected in the absence of an primary antibody.

### Apoptosis detection after PGF_2α _treatment of pCL-MVEC

**Figure 6:**
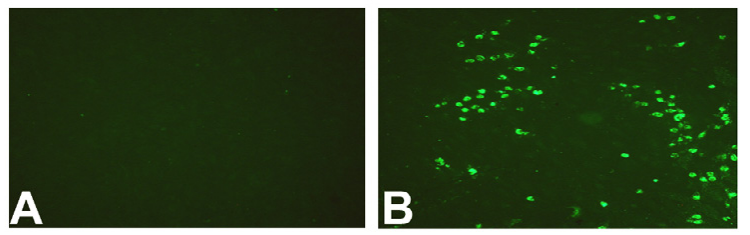
Effect of PGF_2α _treatment on apoptosis induction in porcine corpus luteum-microvascular endothelial cells (pCL-MVECs). A) Representative images of a TUNEL assay in PGF_2α_-treated mid-luteal phase (ML-p) pCL-MVECs. B) Positive TUNEL control (LPS-treated pAECs) (×100).

PGF_2α _treatment failed to induce apoptosis in all pCL-MVEC and pAEC cultures; 30% of the positive controls (LPS-treated pAECs) showed apoptotic nuclei (Fig. 6).

## Discussion

In this study, we describe a reliable method for isolating and setting up primary cultures of pCL-MVECs. A positive selection with the employment of anti-CD-31-coupled magnetic beads was carried out twice. In order to support the attachment of MVECs, a basal medium optimized for bovine lung-derived MVECs supplemented with growth factors (hEGF, VEGF, hFGF, Hydrocortisone) was used until day 4 of the culture. Rare fibroblasts and parenchymal cells were observed in the culture before applying the second positive selection procedure. Cells reached confluence in a specific medium for human endothelial cells supplemented with 15% FBS. The time required to reach confluence varied for each pCL-MVEC culture; however, we observed that EL-p-derived pCL-MVECs proliferated faster than the others. This was in agreement with the high mitotic index of ECs observed during CL formation [40-42]. Confluent pCL-MVEC monolayers showed a heterogeneous morphology as documented in bovine CL-MVECs [23,28]. Various cell phenotypes were present with a predominance of cobble-stone-like and spindle-shaped cells in all pCL-MVECs without any relationship between the morphological types and CL phases analyzed, as reported for bovines [39]. The endothelial origin of the cells was confirmed by traditional endothelial cell marker expression. Moreover, the absence of StAR mRNA in the EC cultures indicated the absence of steroidogenic cell contamination in the confluent MVEC cultures.

FPr expression in CL tissues was found in all the stages analyzed with an increasing trend from the EL- to the ML- phase reaching the maximum during pregnancy, both at mRNA and protein levels in agreement with data already published for the porcine [3] and the bovine species [43]. Our study documents, for the first time, the presence of the FPr on MVECs isolated from swine corpora lutea. We observed that the degree of expression of FPr mRNA varied among the pCL-MVEC cultures in relation to the different CL phases, the highest in the EL-p, decreasing in the ML-p and reaching its lowest level in the P-phase. At the protein level, the presence of the FPr was evidenced in all EC cultures. The amount of FPr protein was very similar among the MVECs derived from ML-p and P-p CLs and pAECs, and was significantly higher on MVECs derived from EL-p CLs. Our data, showing the presence of the FPr on MVECs at early stages testify to the fact that the refractoriness in this species was not be related to the absence of the receptor and rather that other mechanisms must be considered.

The FPr expression level in freshly isolated, overnight and long-term cultured MVECs, was gradually reduced reaching its minimum value at the confluent stage, in agreement with a recent study [31] showing that ECs modify their phenotypes when removed from the microenvironment. Nevertheless, in our model, steroid supplementation (P_4 _and E_2_) resembling the in vivo hormonal environment was not able to regulate FPr expression. Further studies may be required to establish if and how culture conditions may influence FPr expression as well as to investigate if the receptor is functional on pCL-MVECs. The FPr presence on porcine MVECs is in agreement with data obtained for bovine CLs [7] and contrast with those obtained for swine CLs in an in situ mRNA study [8], thus confirming the hypothesis that the discrepancies among these studies are related to the different techniques used.

Finally, the immunolocalization of the FPr on MVECs showed positive staining at plasma membrane level as well as a diffuse perinuclear and cytoplasmatic localization in all cultures. Perinuclear FPr immunoreactivity may be an interaction between the outer membrane of the nuclei and the endoplasmic reticulum where nascent FP protein is formed before being transported to the plasma membrane. The perinuclear staining of P-p CL-MVECs was more intense than others, suggesting an intensive synthesis/glycosilation of the receptor. All these findings are consistent with the distribution of the FPr on ECs of human ocular tissue [44] and on the rat myometrium [45] as well as other prostaglandin receptors on porcine cerebral endothelial cells [46].

In our model, PGF_2α _failed to induce an apoptotic response in all pCL MVEC cultures, in agreement with other in vitro studies conducted on bovines [30,47]. These results contrast with *in vivo *experiments which indicated endothelial cell apoptosis as an early hallmark of PGF_2α_-induced luteal regression in many species [5,6,48]. Therefore, we can affirm that PGF_2α _does not directly induce apoptosis of porcine MVECs in our in vitro model, but the involvement of other molecules should be investigated [47,49,50].

## Authors' contributions

All the authors participated in the experimental design and collected biological material. AZ, CB and TR isolated and optimized the MVEC cultures. CB and TR carried out characterization of the MVECs. AZ and LAR carried out RNA extraction and real-time RT-PCR. CB performed the Western blots. MF performed the immunostaining of the MVECs, apoptosis and statistical analysis. MLB was responsible for animal care and surgical procedures. MLB conceived and supervised the study. AZ, MF and MLB wrote the manuscript. All authors read and approved the final manuscript.
